# Massive Dirac Fermion Observed in Lanthanide-Doped Topological Insulator Thin Films

**DOI:** 10.1038/srep15767

**Published:** 2015-10-27

**Authors:** S. E. Harrison, L. J. Collins-McIntyre, P. Schönherr, A. Vailionis, V. Srot, P. A. van Aken, A. J. Kellock, A. Pushp, S. S. P. Parkin, J. S. Harris, B. Zhou, Y. L. Chen, T. Hesjedal

**Affiliations:** 1 Department of Physics, Clarendon Laboratory, University of Oxford, Oxford, OX1 3PU, United Kingdom; 2 Department of Electrical Engineering, Stanford University, Stanford, California 94305, USA; 3 Geballe Laboratory for Advanced Materials, Stanford University, Stanford, California 94305, USA; 4 Stuttgart Center for Electron Microscopy, Max Planck Institute for Intelligent Systems, Heisenbergstr. 3, 70569 Stuttgart, Germany; 5 IBM Almaden Research Center, 650 Harry Road, San Jose, California 95120, USA; 6 Advanced Light Source, Lawrence Berkeley National Laboratory, Berkeley, California 94720, USA

## Abstract

The breaking of time reversal symmetry (TRS) in three-dimensional (3D) topological insulators (TIs), and thus the opening of a ‘Dirac-mass gap’ in the linearly dispersed Dirac surface state, is a prerequisite for unlocking exotic physical states. Introducing ferromagnetic long-range order by transition metal doping has been shown to break TRS. Here, we present the study of lanthanide (Ln) doped Bi_2_Te_3_, where the magnetic doping with high-moment lanthanides promises large energy gaps. Using molecular beam epitaxy, single-crystalline, rhombohedral thin films with Ln concentrations of up to ~35%, substituting on Bi sites, were achieved for Dy, Gd, and Ho doping. Angle-resolved photoemission spectroscopy shows the characteristic Dirac cone for Gd and Ho doping. In contrast, for Dy doping above a critical doping concentration, a gap opening is observed via the decreased spectral intensity at the Dirac point, indicating a topological quantum phase transition persisting up to room-temperature.

Doping of the prototypical 3D-TIs^1^,^2^ (Bi,Sb)_2_(Se,Te)_3_ with transition metal ions can lead to ferromagnetic ordering at low temperatures^3^,^4^. The major challenge to realizing the quantum anomalous Hall effect (QAHE) in magnetic TIs is to simultaneously achieve low bulk carrier densities and a Dirac-mass gap^5^. Cr doping has been the key for observing the QAHE^6^,^7^. While QAHE studies on Cr:(Bi,Sb)_2_Te_3_ were carried out at mK temperatures, the magnetic transition temperature is 15 K (ref. 6). However, recent studies on this materials system have found extreme dopant-induced Dirac-mass disorder^8^. The need to raise the transition temperature and the size of the gap for any kind of practical application, combined with the need to prevent disorder in the system introduced by doping, motivated us to explore the introduction of the highest magnetic moments available in the periodic table of elements.

The lanthanide (Ln) 4*f* series comprises the elements from La to Yb, which are most commonly found in a +3 oxidation state, allowing for an isoelectronic substitution of Bi. The magnetic moment of Gd^3+^, which has the maximum number of 7 unpaired *f* electrons in the series, is 7.94 *μ*_B_. However, even higher moments of ~10.6 *μ*_B_ are found for Dy^3+^ (4*f*^  9^) and Ho^3+^ (4*f*^ 10^) due to spin-orbit coupling. We explored Ln doping of Bi_2_Te_3_ with all three elements: Dy, Ho, and Gd. Gd- and Ho-doped films do not show a gap opening and we refer to refs 9, 10, 11 for details. Dy doping, on the other hand, shows intriguing results which are the focus of this letter.

A series of (Dy_*x*_Bi_1−*x*_)_2_Te_3_ thin films (0 ≤ *x* ≤ 0.355) was grown on *c*-plane sapphire substrates by molecular beam epitaxy (MBE). *In situ* reflection high-energy electron diffraction (RHEED) was used to provide qualitative information about the surface morphology during film growth (cf. Fig. 1a). Streak-like diffraction patterns, indicative of a flat, crystalline film surface, were observed for all samples up to and including *x* = 0.183, above which the streaks became more diffuse, indicative of a rougher surface. In general, the surface morphology is dominated by triangular domains with a terrace-step structure, however, with increasing doping level, the density of seeds and subsequent triangular structures increases as shown in Fig. 1b.

The elemental compositions as well as the thicknesses of the thin films were determined from a combination of Rutherford backscattering spectrometry (RBS) and particle induced x-ray emission (PIXE), as gettering effects prevented beam flux monitoring of the Dy flux. All films showed evidence for small amounts of unintentional Se doping, however, the combined Te and Se atomic percentages are ~60%. The cation (Dy + Bi) to anion ratio is ~2:3, within the specified error margin, which is indicative of Dy being mostly substitutional on Bi sites^9^. However, at the highest doping level (*x* = 0.355), the anion atomic percentage was found to be slightly less than 60% which could indicate a mixed substitutional and interstitial doping scenario^9^. No signs of compositional gradients or phase segregation were observed in RBS/PIXE for Dy cation substitution up to 35.5%.

To investigate the structural properties of the films x-ray diffraction (XRD) studies were performed. Figure 1c shows symmetric 2*θ*–*ω* scans. For all samples, only substrate and film peaks with relative positions consistent with the (0 0 3*l*) family of Bi_2_Te_3_ diffraction peaks were observed. This indicates that, even at large Dy concentrations, the films are *c*-axis oriented and grown in a rhombohedral crystal structure. Secondary phases were not observed for concentrations up to *x* ≤ 0.355. The doping levels achieved in this study were remarkably high given that the two binary end members, Bi_2_Te_3_ and Dy_2_Te_3_, possess different crystal structures (rhombohedral and orthorhombic, respectively) which may cause the doped system to be more susceptible to phase segregation. Phase segregation is commonly reported in magnetically doped TI systems, often at lower doping concentrations than were achieved in this study^12^. Peak broadening and intensity variations, indicative of degradation in crystalline quality, were observed particularly for high-angle reflections (2*θ* > 65° in Fig. 1c) with increasing doping concentration^9^,^13^. Rocking curves of the (0 0 15) diffraction peaks, as shown in Fig. 1c, confirm this trend. Films with Dy concentrations *x* ≤ 0.113 exhibit a very low degree of mosaicity (full width at half maximum (FWHM) <0.5°).

With increasing Dy concentration, the peak positions (Fig. 1c) shift towards lower diffraction angles, i.e., the *c*-axis lattice constant increases as shown in Fig. 2b (centre plot). The *c*-axis lattice constants vary from (30.39 ± 0.02) Å for the undoped Bi_2_Te_3_ film to (30.83 ± 0.05) Å for the highest concentration investigated (*x* = 0.355). The literature values for the in-plane and out-of-plane lattice parameters for rhombohedral DyBiTe_3_ (i.e., *x* = 0.5 in (Dy_*x*_Bi_1−*x*_)_2_Te_3_)) are *a* = 4.14 Å and *c* = 31.02 Å, respectively^14^. When compared to the lattice parameters for undoped Bi_2_Te_3_^14^, DyBiTe_3_ has a smaller in-plane lattice constant (5.8% decrease) and a larger out-of-plane lattice constant (1.9% increase). Therefore, the trends observed in lattice parameters as a function of Dy doping (see Fig. 2b, top panel, for *a*-axis lattice constants) are consistent. Note that the *x* = 0.355 doping condition shows deviations in the general trends observed for the in-plane lattice parameter and unit cell volume for the doping series, which probably indicates onset of mixed substitutional and interstitial doping, while the other samples are purely substitutionally doped.

Further investigations of the structural properties were performed using 2D reciprocal space mapping (RSM). Figure 2 shows selected RSMs for the doping series. The red lines, passing through to the centremost position of the 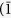 0 20) peak for the undoped sample, are used as guides to aid the visualisation of changes in the peak positions. As shown in Fig. 2b, the out-of-plane lattice parameter, determined from the *Q*_*y*_ values of the centremost positions of the (Dy_*x*_Bi_1−*x*_)_2_Te_3_ (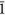 0 20) peaks, continuously increases as a function of doping which is consistent with the results from the symmetric XRD scans. Slight variations in the in-plane lattice parameter, determined by *Q*_*x*_ positions of the 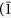 0 20) peaks, were also found within the doping series and are shown in Fig. 2b. The calculated unit cell volumes, determined from the RSM determined *a*- and *c*-axis lattice constants, are provided in Fig. 2b. For samples with *x* ≤ 0.113, the 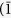 0 20) peaks exhibit a prominent elongation along the *Q*_*y*_ direction which may indicate the presence of either microstrain or nonuniform doping throughout the layer thickness^15^. For higher doping concentrations, the more oval peak shape suggests a higher dislocation density in the film and higher degree of mosaicity. For all doping concentrations, an elongation along the *Q*_*x*_ direction was observed which is attributed to any combination of finite size effects of the domains, tilting at domain boundaries (i.e., mosaicity), as well as interface defects^16^.

Scanning transmission electron microscopy (STEM) was used to provide further insight into the structure of Dy-doped thin films. A high-angle annular dark field (HAADF)-STEM image of a (Dy_0.113_Bi_0.887_)_2_Te_3_ film acquired at 60 kV is shown in Fig. 3a. The characteristic crystal structure formed by the stacked quintuple layers separated by van der Waals gaps is clearly resolved. Energy-dispersive x-ray (EDX) line scans were acquired traversing the van der Waals gap between adjacent quintuple layers (see orange arrow in Fig. 3a). The corresponding profiles of Bi-M, Te-L and Dy-L x-ray emission intensities along the line scan displayed together with the HAADF-STEM intensity profile are presented in Fig. 3b. The Dy-L signal indicates the substitutional incorporation of Dy atoms on Bi sites and the absence of Dy in the van der Waals gaps. An EDX line scan obtained along the Bi lattice planes is shown in the Supplementary Fig. 1. No evidence for clusters or local phase segregation were detected by the HAADF-STEM and EDX investigations, which supports the presented XRD analysis.

Angle-resolved photoemission spectroscopy (ARPES) was used to probe the electronic band structure. The breaking of TRS can be detected by a gap opening at the Dirac point (DP) in the topological surface state (TSS) band. In undoped Bi_2_Te_3_ a strong spectral intensity is observed at the DP in the TSS band. This is a manifestation of Kramer’s degeneracy which indicates the presence of TRS in the system. Without TRS breaking, the TSS band remains two-fold spin-degenerate at the DP, even if the system is perturbed by non-magnetic dopants^4^. However, if the TRS is broken by ferromagnetic ordering, the TSS band dispersion becomes discontinuous and the spectral intensity at the DP is suppressed which indicates the presence of a gap^4^.

Figure 4 shows band structures obtained along the Γ-*K* direction for two doping concentrations. Similar to the surface state observed on undoped Bi_2_Te_3_, ‘V’-shaped surface states with linear dispersions exist on samples with *x* ≤ 0.055 (see Fig. 4a). Strong spectral intensities were also found at the DPs for samples with *x* ≤ 0.055 which indicates continuity of the TSS band and the presence of TRS. However, for *x* = 0.113 shown in Fig. 4b, the TSS band dispersion appears to follow the characteristic ‘V’-shape near the Fermi Level but deviates near the Dirac point, where a reduction in the spectral intensity is observed.

Energy distribution curves (EDC) at 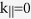 were obtained to investigate the reduced spectral intensity and to explore the possibility of a magnetic doping induced gap, which are shown on the right-hand side of Fig. 4a–d. The measurement temperature for Fig. 4a,b was 20 K, whereas Fig. 4c,d were obtained at 300 K. Figure 4a,c for *x* = 0.055, show continuity of the TSS band by the single-peak structure of the EDCs^4^,^17^. However, in Fig. 4b,d for *x* = 0.113, a dip or twin-peak structure around the Dirac point is observed in the EDC plots which suggests that the TSS band dispersion is gapped. From the analysis of the EDC plots a gap size of ~85 meV is estimated. On the other hand, no gaps were observed for Gd- and Ho-doped Bi_2_Te_3_ thin films^9^,^11^.

Long-range ferromagnetic order has not been detected in rare earth-doped Bi_2_Te_3_ thin films in previous magnetic properties studies down to a measurement temperature of 2 K (refs 9, 10, 11, 17, 10, 11). Evidence for a gapped TSS band in the absence of detectable long-range ferromagnetic order in the bulk is an unexpected but not unprecedented phenomenon in magnetically doped TIs^19^,^20^. Recently, Chang *et al*.^19^ reported the existence of a gapped TSS band in ARPES measurements on Bi_2−*x*_Cr_*x*_Se_3_ thin films without indication of long-range ferromagnetic order in direct magnetization measurements down to 1.5 K, which was attributed to short-range ferromagnetic order induced by inhomogeneous magnetic doping and the formation of Cr clusters. It has also been suggested that impurity scattering may play a more significant role than previously considered^21^,^22^. This paradox is highlighted further by comparing theoretical and experimental studies on Mn-doped Bi_2_Te_3_ and Bi_2_Se_3_ systems which have experimentally detected gaps up to an order of magnitude larger than theoretically predicted values and persist well above the Curie temperatures^18^,^23^. It is important to note, however, that disorder alone is an unlikely cause of the observed gap as it is only observed for Dy-doped films, whereas Ho and Gd doping, which leads to very similar structural and magnetic properties, fails to open up a gap^9^,^10^,^11^. Further experimental studies using spin-resolved ARPES may provide conclusive evidence of the existence of the topological phase in the Dy-doped samples^24^,^25^. Accompanying first-principles calculations are also urgently needed, which are complicated by the highly correlated, atomic-like nature of the 4*f* electrons in Ln systems^26^. In light of these theoretical challenges, only exploratory materials science can tell whether a Ln doped system behaves as expected, or holds unforeseen surprises.

## Methods

### Thin film growth

(Dy_*x*_Bi_1−*x*_)_2_Te_3_ thin films were grown on *c*-plane sapphire substrates by co-evaporation of high purity Bi (6N), Te (6N), and Dy (4N) from standard Knudsen effusion cells in an MBE system (Createc GmbH, Erligheim, Germany). Cell fluxes were calibrated using an ion gauge beam flux monitor. The Dy concentration, *x*, was controlled by varying the Dy effusion cell temperature while the Bi and Te cell temperatures were held constant yielding a nominal Te/Bi ratio of 15. The typical growth rate was ~12 Å/min. Similar parameters were used for the growth of Ho- and Gd-doped films^9^,^10^,^11^.

Cleaned *c*-plane sapphire substrates were introduced into the main growth chamber with a base pressure of ~5 × 10^−11^ Torr for a short anneal at 450 °C. Thin film deposition was carried out using a two-temperature step growth process similar to the one established for undoped films^27^. During the first growth step, a low temperature nucleation layer was deposited for 33 min at 250 °C. Film growth was then paused while the substrate temperature was ramped up to 300 °C under Te flux at a rate of 5 °C/min. At 300 °C, the sample was annealed for 30 min before growth was then continued until the desired thickness was achieved. RHEED was used for *in situ* monitoring of the film growth process on selected samples. The surface morphology was inspected *ex situ* using an AFM.

### Elemental characterisation

The elemental compositions, as well as the film thicknesses, were determined from a combination of RBS using 2.3 MeV helium ions and PIXE using 1 MeV hydrogen ions. PIXE analysis was required in order to resolve an overlap of the Ln signal with the Bi and Te signals in the RBS measurements. By fitting the combined RBS and PIXE data to structural models, the reported elemental compositions and film thicknesses were determined.

### X-ray based structural analysis

XRD measurements were performed using a Bruker D8 Discover x-ray diffraction system with Cu K_*α*1_ emission to investigate the effects of Dy incorporation on the host Bi_2_Te_3_ crystalline structure. For asymmetric 2D reciprocal space mapping, a PANalytical X’Pert PRO system with a 1D PIXcel detector in grazing exit configuration was used. For all scans, the diffractometer was aligned to the Al_2_O_3_ 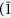 0 8) peak and scanned over the relative locations of the Bi_2_Te_3_ 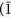 0 19) and 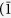 0 20) peaks.

### TEM lamellae preparation and scanning transmission electron microscopy

High-quality samples with a Dy concentration of *x* ≤ 0.113 were selected for HAADF-STEM and EDX-STEM investigations. The (Dy_0.113_Bi_0.887_)_2_Te_3_ film was carefully delaminated from the substrate using a droplet of glue. Afterwards, thin TEM lamellae were cut of the delaminated films in cross-sectional geometry using an ultramicrotome (Leica EM-UC6, Leica microsystems, Wetzlar, Germany). Thin sections were obtained using an 35° water-filled diamond knife (Diatome, Biel, Switzerland) to cut approximately 40 nm thick slices that were captured on Cu grids covered with a lacey carbon film.

HAADF-STEM and EDX measurements were carried out at 60 kV and 200 kV with an advanced analytical TEM/STEM, JEOL ARM200F (JEOL Co. Ltd), equipped with a cold field-emission gun and a DCOR probe Cs corrector (CEOS Co. Ltd.). Elemental profiles were obtained by acquiring EDX line scans using a 100 mm^2^ JEOL Centurio SDD-EDX detector (JEOL Co. Ltd.) and the Thermo Noran System 7 EDX system (Thermo Fisher Scientific Inc.).

All TEM data were analysed using the commercial software Digital Micrograph (Gatan Inc.). For noise removal from the HAADF-STEM images and the EDX line scans a commercially available software package using multivariate statistical analysis (MSA) for Digital Micrograph from HREM Research Inc., a script function written by D.R.G. Mitchell, were used.

### Angle-resolved photoemission spectroscopy

ARPES measurements were performed at beamline 10.0.1 of the Advanced Light Source (ALS) at Lawrence Berkeley National Laboratory. The measurement pressure was kept below 3 × 10^−11^ Torr at all times. The data was recorded using a Scienta R4000 analyser at variable sample temperatures. The total convolved energy and angle resolutions were 16 meV and 0.2°, respectively. The fresh surface for ARPES measurement was obtained by cleaving the thin film sample *in situ* as described in ref. 28.

## Additional Information

**How to cite this article**: Harrison, S. E. *et al*. Massive Dirac Fermion Observed in Lanthanide-Doped Topological Insulator Thin Films. *Sci. Rep*. **5**, 15767; doi: 10.1038/srep15767 (2015).

## Supplementary Material

Supplementary Information

## Figures and Tables

**Figure 1:**
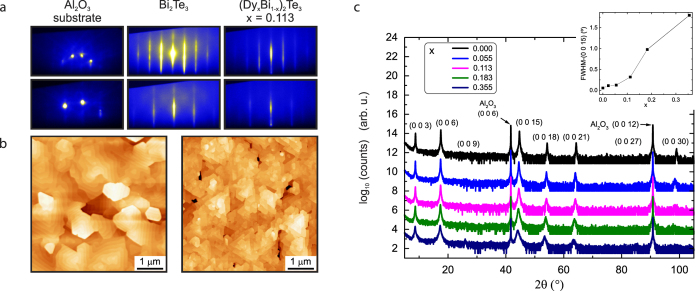
Surface morphology and structural properties. (**a**) Sequence of RHEED images of the substrate, an undoped (Bi_2_Te_3_) film, and a Dy-doped film with *x* = 0.113 for comparison. Diffraction patterns obtained along the [10
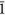
0] azimuth (top) and [11
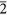
0] azimuth (bottom) of *c*-plane sapphire occur upon 30° rotation, which reflects the three-fold symmetry of the crystal^29^. (**b**) Atomic force microscopy images of an undoped Bi_2_Te_3_ film and Dy-doped film with *x* = 0.055. With increasing Dy doping level, but *x* ≤ 0.055, the morphology stays similar, however, the density of seeds and subsequent triangular structures increases as shown on the right. For samples with *x* ≥ 0.113, the characteristic concentric hillock-like growth spirals are no longer the prominent feature. The surface roughness was also found to increase with doping which is consistent with the observed RHEED patterns. (**c**) 2*θ*-*ω* scans and the FWHM of the (0 0 15) rocking curves, shown as an inset, as a function of Dy doping concentration. The FWHM values increase from 0.0555° for the undoped case to 1.7961° for the highest doping concentration, i.e., the Dy-doped films are of higher quality than Cr-doped Bi_2_Se_3_ thin films^30^.

**Figure 2:**
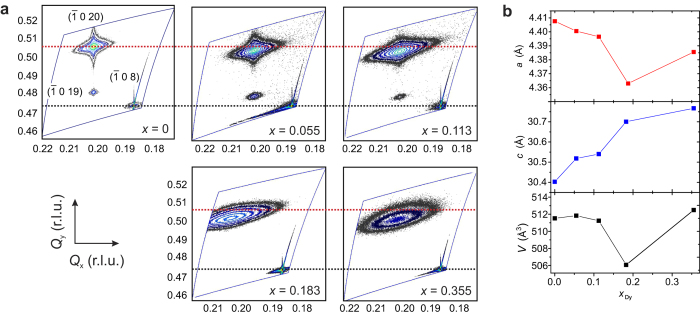
Reciprocal space mapping. (**a**) Scans were obtained by aligning to the Al_2_O_3_ 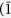 0 8) peak and scanning over the relative positions of the Bi_2_Te_3_ 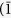 0 20) and 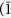 0 19) peaks for *x* = 0, 0.055, 0.113, 0.183, and 0.355 as indicated. For all samples, a misalignment of the substrate and film peaks in *Q*_*x*_ is observed, as expected from an incoherent growth on Al_2_O_3_. (**b**) Lattice constants and unit cell volume as a function of Dy doping concentration. From top to bottom: in-plane lattice parameter (*a*-axis), out-of-plane lattice parameter (*c*-axis), and unit cell volume *V* determined from the RSM 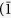 0 20) peak positions.

**Figure 3:**
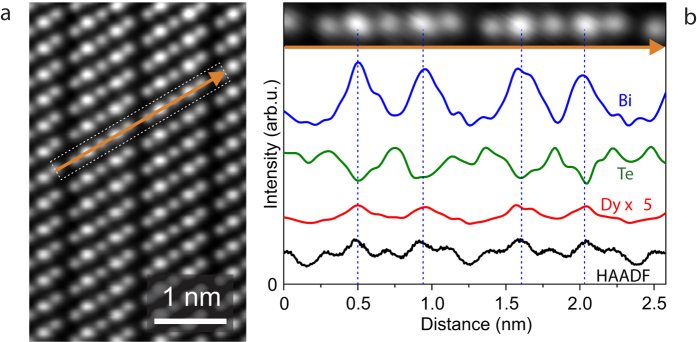
Structural and chemical mapping. (**a**) HAADF-STEM image of a (Dy_0.113_Bi_0.887_)_2_Ti_3_ film with marked position of measured EDX line scan (orange arrow) across the van der Waals gap. (**b**) Corresponding profiles of Bi-M (blue), Te-L (green) and Dy-L (red) x-ray emission intensities along the EDX line scan together with the HAADF-STEM intensity profile (black). No Dy signal is detectable in the gap.

**Figure 4:**
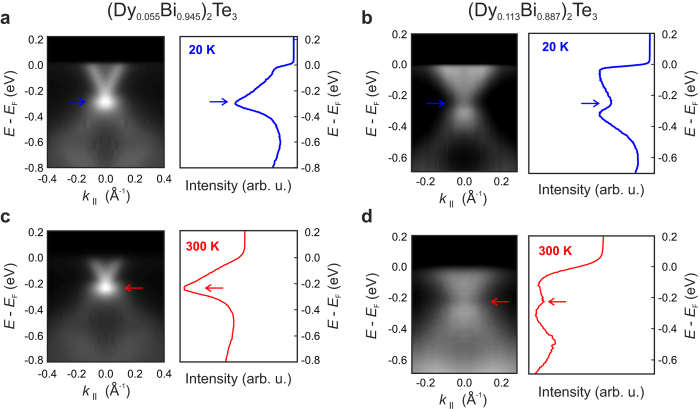
Angle-resolved photoemission spectroscopy of low and highly doped films. Band structures obtained along the *K*–Γ–*K* direction and energy distribution curves along 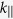 = 0 for Dy-doped samples with (**a,c**), *x* = 0.055 and (**b,d**), *x* = 0.113, measured at 20 K and 300 K, respectively. Note that the arrows indicate the relative locations of the Dirac points in the band structure and energy distribution curves. The measurements reveal strong spectral intensities at the DPs in the TSS up to 300 K for samples with *x* ≤ 0.055, however, for samples with *x* = 0.113, a gap on the order of ~85 meV was consistently detected in band structure and EDC plots. These sizeable gaps persist in measurements performed at room temperature.
