# Supplementary material for: Massive Dirac Fermion Observed in Lanthanide-Doped Topological Insulator Thin Films
Source: Sci Rep. 2015 Oct 27;5:15767. doi: 10.1038/srep15767 (PMC4621505; doi:10.1038/srep15767)
Supplement: Supplementary Information [file srep15767-s1.pdf]

Supporting Online Material for

Massive Dirac Fermion Observed in Lanthanide-Doped Topological Insulator Thin Films

S. E. Harrison,[1,2] L. J. Collins-McIntyre,[1] P. Schönherr,[1] A. Vailionis,[3] V. Srot,[4] P. A. van Aken,[4] A. J. Kellock,[5] A. Pushp,[5] S. S. P. Parkin,[5] J. S. Harris,[2] B. Zhou,[1,6] Y. L. Chen,[1] and T. Hesjedal[1*]

[1]Department of Physics, Clarendon Laboratory, University of Oxford, Oxford, OX1 3PU, United Kingdom

[2]Department of Electrical Engineering, Stanford University, Stanford, California 94305, USA

[3]Geballe Laboratory for Advanced Materials, Stanford University, Stanford, California 94305, USA

[4]Stuttgart Center for Electron Microscopy, Max Planck Institute for Intelligent Systems, Heisenbergstr. 3, 70569 Stuttgart, Germany

[5]IBM Almaden Research Center, 650 Harry Road, San Jose, California 95120, USA

[6]Advanced Light Source, Lawrence Berkeley National Laboratory, Berkeley, California 94720, USA

HAADF-STEM image and EDX line scans obtained along the Bi lattice planes is shown in the Supplementary Figure 1.

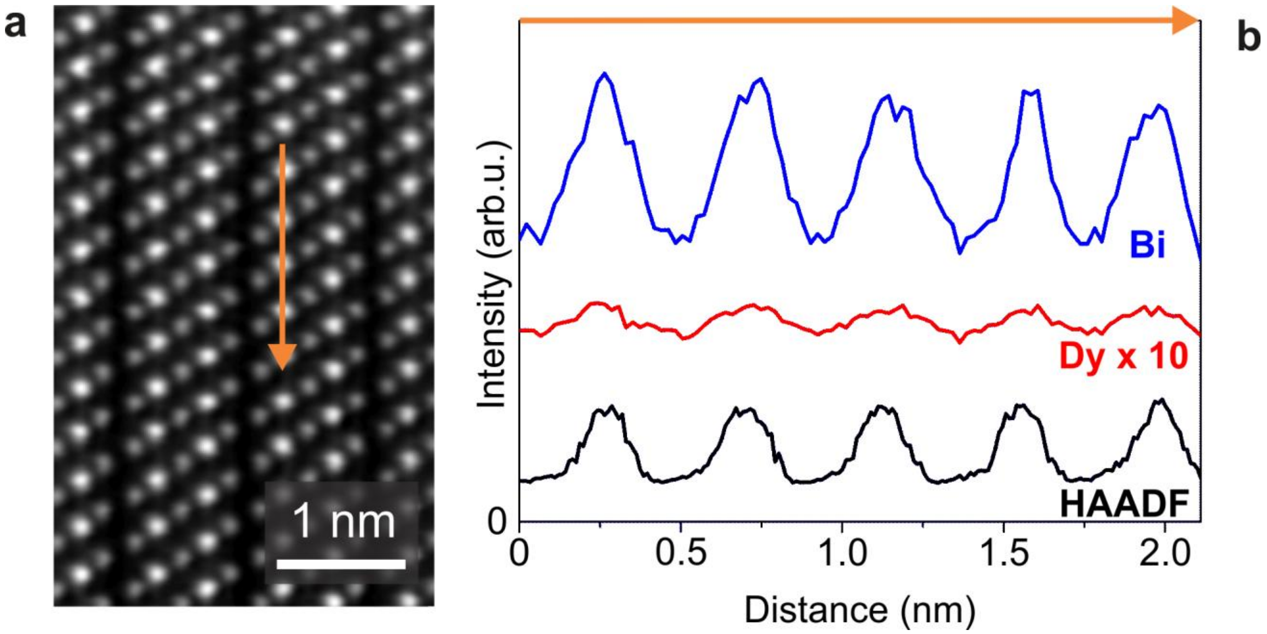

Figure S1. **a,** HAADF-STEM image of a $(Dy_{0.113}Bi_{0.887})_2Te_3$ film with marked position of measured EDX line scan (orange arrow). **b,** Corresponding profiles of Bi-M (blue) and Dy-L (red) x-ray emission intensities along the line scan together with the HAADF-STEM intensity profile (black).
